# A High-Precision Method for Dynamically Measuring Train Wheel Diameter Using Three Laser Displacement Transducers

**DOI:** 10.3390/s19194148

**Published:** 2019-09-25

**Authors:** Fajia Zheng, Bin Zhang, Run Gao, Qibo Feng

**Affiliations:** Key Lab of Luminescence and Optical Information, Ministry of Education, Beijing Jiaotong University, Beijing 100044, China; 16118441@bjtu.edu.cn (F.Z.); bzhang@bjtu.edu.cn (B.Z.); 17118454@bjtu.edu.cn (R.G.)

**Keywords:** train wheel diameter measurement, dynamic measurement, laser displacement transducer, structural light vision transducer, multi-body dynamics and finite element methods

## Abstract

Wheel diameter is a significant geometric parameter related to the safe operation of trains, and needs to be measured dynamically. To the best of the authors’ knowledge, most existing dynamic measurement methods and systems do not meet the requirement that the wheel diameter measurement error for the high-speed vehicle is less than 0.3 mm. In this paper, a simple method for dynamically and precisely measuring train wheel diameter using three one-dimensional laser displacement transducers (1D-LDTs) is proposed for the first time, and a corresponding measurement system which was developed is described. The factors that affect the measurement accuracy were analyzed. As a main factor, rail deformation caused by the wheel-rail interaction force at low (20 km/h) and high (300 km/h) speeds was determined based on the combination of multi-body dynamics and finite element methods, and the effect of rail deformation on measurement accuracy is greatly reduced by a comparative measurement. Field experiments were performed to verify the performance of the developed measurement system, and the results of the repeatability error and measurement error of the system were both less than 0.3 mm, which meets the requirement of wheel diameter measurements for high-speed vehicles.

## 1. Introduction

Wheelsets are a key component of trains. The wear of wheel treads is becoming more serious, due to continuous increases in the speed and load of trains [1]. As wheel diameter is a significant geometric parameter related to the safe operation of trains, the diameter differences between the wheels of one wheelset, one bogie, one car, and one train must be controlled under a safe limitation. For example, the diameter difference within one wheelset of the high-speed vehicle in operation must be less than 0.3 mm, according to European Norm EN 15313 [2]. The wheel diameter changes every day because of wheel wear; thus, the dynamic measurement of diameter to detect overrun wheels in time is critically important.

Currently, there are many kinds of wheel diameter measurement methods and devices, which can be classified into two categories: shop measurements and field measurements. Shop measurements are usually performed periodically in the train garage. Traditionally, a measuring gauge [3] is utilized to manually measure the diameter based on the three-point measurement method. It is unreliable, as the skill of the user can significantly influence the result. To improve the measuring reliability, Torabi et al. proposed a method based on image processing with a measurement uncertainty of 0.4 mm [4]. Feng et al. proposed a method which uses parallelogram mechanisms together with one-dimensional laser displacement transducers (1D-LDTs) [5]. A non-contact measurement method using the LDT and CCD was put forward by Wu et al. [6] with a measuring accuracy of 0.2 mm. The underfloor wheelset lathe can be used in geometric parameter measurements of the wheel tread with the wheel remaining assembled to the bogie [7]; the accuracy of measuring the diameter is within 0.1 mm. The main disadvantage of shop measurement methods is that a potential safety hazard exists, because these methods cannot identify overrun wheelsets in time. Filed measurement is consequently the only choice for the timely discovery of overrun wheelsets. 1D-LDT and structural light vision transducers (SLVTs) are the two main methods for field diameter measurements. Chen et al. used commercial SLVTs to measure the geometric parameters of wheel tread, including wheel diameter [8,9]. Similar measurement systems were developed by corporations [10,11,12]. These methods used multiple sets of transducers which required global calibration on-site to unify the coordinated system; this approach is occasionally difficult, and the diameter measurement error is about 0.5 mm, which cannot meet the requirement of being less than 0.3 mm for the wheels of a high-speed vehicle, according to Chinese Standards [13]. The measurement accuracy of diameter can be improved by 1D-LDT, as the 1D-LDT has higher measurement accuracy. Naumann et al. proposed a measurement system using at least 26 1D-LDTs [14], which is difficult to apply on-site. We propose a series of methods using 1D-LDTs [15,16]. One 1D-LDT was used to dynamically measure the diameter and an eddy current sensor was used to locate the position of the wheel [15]. In order to improve the accuracy of the positioning, two eddy current sensors were used [16]. However, the measurement accuracy of these methods will be significantly reduced due to wear of the wheel tread and rail top. To solve this problem, we further proposed a simple method using three 1D-LDTs. The corresponding measurement system was developed, and field experiments were carried out. The results show that the repeatability error and measurement error are less than 0.3 mm, which meet the requirement of Chinese high-speed railway operations [13], and pave a new way for dynamically measuring wheel diameters with greater precision. The rest of this paper is organized as follows. The proposed method is described in Section 2. Section 3 analyses the factors that affect the measuring accuracy. In Section 4, the results of field experiments are presented. Finally, the conclusions of this study are given in Section 5.

## 2. Measurement Method

### 2.1. Measurement Method Based on One 1D-LDT

**Figure 1:**
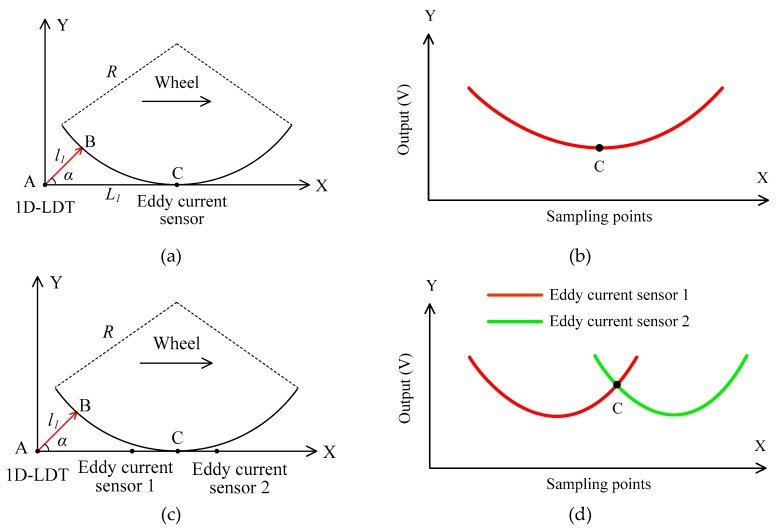
Schematic of measurement methods based on one 1D-LDT: (**a**) one 1D- LDT and eddy current sensor; (**b**) output of the eddy current sensor; (**c**) one 1D-LDT and two eddy current sensors; (**d**) outputs of two eddy current sensors.

Figure 1a shows the method for dynamically measuring the wheel diameter using one 1D- LDT and eddy current sensor [15]. As shown in Figure 1a, the 1D-LDT is fixed on the rail at point A with an angle *α*. When the train wheel runs on the rail along the X axis, the 1D-LDT measures the distance *l*_1_ between the transducer and the wheel tread at the point of B. The coordinate of B is (*l*_1_*cosα*, *l*_1_*sinα*). An eddy current sensor is mounted at point C to measure the wheel position, which is *L*_1_ away from point A. In fact, the eddy current sensor can be replaced by 1D-LDT. Thus, the equation of the circle is expressed as

(1) $${\left(x-L_{1}\right)}^{2}+{\left(y-R\right)}^{2}=R^{2}.$$

where, *R* is the radius of the wheel. According to the measured distance *l*_1_ of the 1D-LDT when the wheel is in the position of point C, the diameter is calculated by

(2) $$D=\frac{L_{1}{}^{2}+l_{1}{}^{2}}{l_{1}\sin\alpha }-\frac{2L_{1}}{\tan\alpha }.$$

The eddy current sensor is used to measure the wheel position; however, the output of the eddy current sensor changes slowly when it is closer to the lowest point of the wheel, as shown in Figure 1b. As a result, the positioning accuracy is low, which greatly influences the accuracy of diameter measurements. To improve the accuracy of the positioning, two eddy current sensors [16] are mounted symmetrically at appropriate positions on both sides of the point C, as shown in Figure 1c, and the two outputs of the eddy current sensors change quickly at the point C, as shown in Figure 1d. There is a zero position of the difference between the two outputs, and this point is used to determine the position of the wheel, thus improving the positioning accuracy and the diameter measurement accuracy.

### 2.2. Measurement Method Based on Two 1D-LDTs 

**Figure 2:**
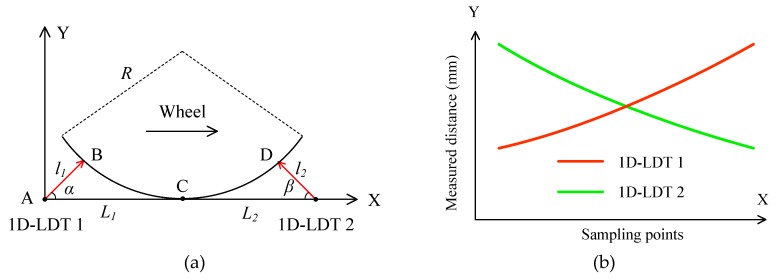

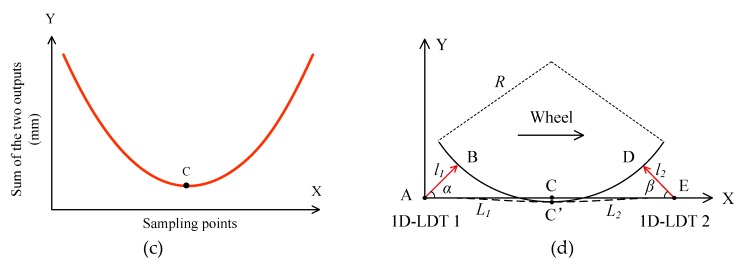
Schematic of measurement methods based on two 1D-LDTs: (**a**) two 1D-LDTs; (**b**) outputs of the two 1D-LDTs; (**c**) sum of the two outputs of 1D-LDTs; (**d**) position change of the point C.

To reduce the influence of roundness on the diameter measurements, two 1D-LDTs were used, as shown in Figure 2a. The second transducer is fixed on the rail at the point E with an angle *β*, which is *L*_2_ away from the point C. When the train wheel runs on the rail along the X axis, two transducers can measure the distances *l*_1_ and *l*_2_ synchronously, and the outputs of the two 1D-LDTs are shown in Figure 2b. Figure 2c shows the sum of the two outputs of 1D-LDTs. From Figure 2c, there is a minimum value which is used to determine the wheel position with a high level of accuracy. Thus, the measured distances *l*_1_ and *l*_2_ of the two 1D-LDTs are determined when the wheel is in the position of point C.

According to the Equation (2), the diameter of wheel is expressed as

(3) $$D=\frac{L_{1}{}^{2}+l_{1}{}^{2}}{2l_{1}\sin\alpha }-\frac{L_{1}}{\tan\alpha }+\frac{L_{2}{}^{2}+l_{2}{}^{2}}{2l_{2}\sin\beta }-\frac{L_{2}}{\tan\beta }.$$

### 2.3. Measurement Method Based on Three 1D-LDTs

The above three methods (in Figure 1a,c, and Figure 2a) are based on the assumption that the point C between the wheel and the rail is fixed. In fact, it will change, due to wear of the wheel tread and rail top. If the point C is changed to C′, as shown in Figure 2d, it will yield a measurement error for the 1D-LDT, thereby significantly reducing the measurement accuracy. To solve this problem, a simple method using three 1D-LDTs is proposed.

**Figure 3:**
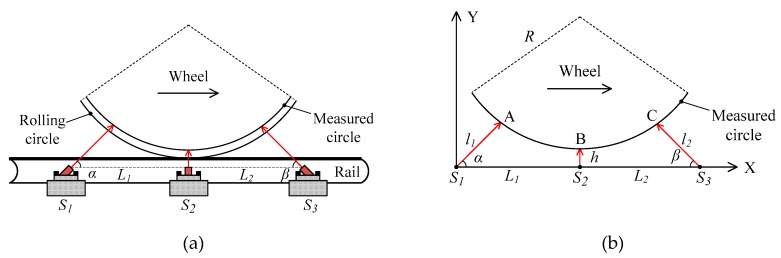
Schematic of the measurement method using three 1D-LDTs: (**a**) setup; (**b**) schematic diagram.

Figure 3a shows the setup for dynamically measuring the diameter using three 1D-LDTs, which are called *S*_1_, *S*_2_, and *S*_3_, respectively. Three transducers are mounted on the standard rail. The distances between *S*_2_ to *S*_1_ and *S*_3_ are *L*_1_ and *L*_2_, respectively. In addition, the emitting spots of *S*_1_ and *S*_3_ are projected to the wheel tread at the angles of *α* and *β*, respectively.

Figure 3b shows the schematic diagram of diameter measurement. From Figure 3b, the coordinate of point A is (*l*_1_*cosα*, *l*_1_*sinα*), and the equation of the measured circle is expressed as

(4) $${\left(x-L_{1}\right)}^{2}+{\left(y-R-h\right)}^{2}=R^{2}.$$

where *R* is the radius of the measured circle. From Equation (4), the diameter of the measured circle *D*_1_ is calculated by

(5) $$D_{1}=\frac{L_{1}{}^{2}+l_{1}{}^{2}+h^{2}-2L_{1}l_{1}\cos\alpha -2l_{1}h\sin\alpha }{l_{1}\sin\alpha -h}.$$

Similarly, the diameter of the circle *D*_2_ measured by *S*_2_ and *S*_3_ can be obtained. To reduce the influence of roundness, the average of the two diameters *D*_1_ and *D*_2_ is the optimal diameter *D_m_* of the measured circle. With *L*_1_ = *L*_2_ = *L* and *α* = *β*, it is expressed by

(6) $$D_{m}=\frac{L^{2}+l_{1}{}^{2}+h^{2}-2Ll_{1}\cos\alpha -2l_{1}h\sin\alpha }{2l_{1}\sin\alpha -2h}+\frac{L^{2}+l_{2}{}^{2}+h^{2}-2Ll_{2}\cos\alpha -2l_{2}h\sin\alpha }{2l_{2}\sin\alpha -2h}.$$

**Figure 4:**
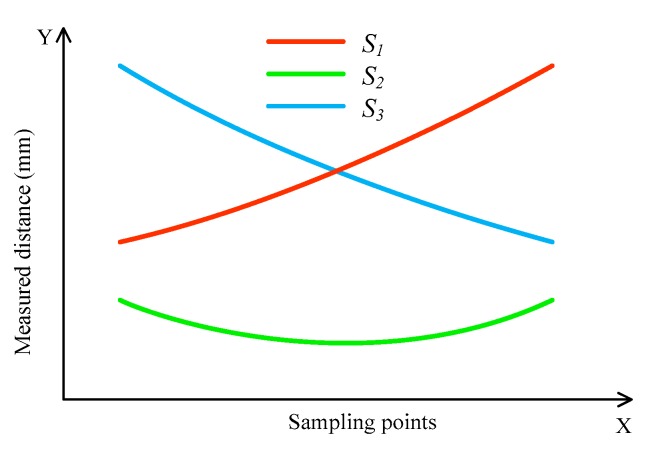
Typical measuring distance of the three transducers.

When the train wheel passes through the measurement system, the three transducers synchronously measure the distance between the transducer and the wheel tread. Figure 4 shows the typical outputs of the transducers. The closest distance *h* between *S*_2_, and the measured circle can be obtained by fitting using the sampling points of *S*_2_. Similar to the method using two 1D-LDTs, the sum of the outputs of *S*_1_ and *S*_3_ is used to determine the wheel position. Then, the measured distances *l*_1_ and *l*_2_ of the other two transducers *S*_1_ and *S*_3_ are obtained, respectively. Finally, the diameter of the measured circle can be determined according to Equation (6). Different from the methods shown in Figure 1a,c, and Figure 2a, *S*_2_ in Figure 3b is used to measure distance *h*, rather than for determining the wheel position. If there is the wear on the wheel tread and rail top, the distance *h*, *l*_1_, and *l*_2_ will be changed correspondingly, and Equation (6) is still stable; thus, the diameter measurement accuracy will not be affected by the wear of the wheel tread and rail top.

**Figure 5:**
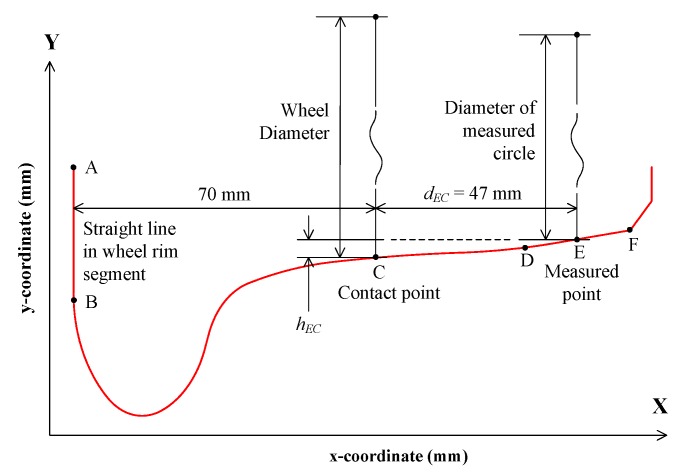
Wheel tread profile.

Figure 5 shows the wheel tread profile. Point C is defined as the contact point between the wheel and the rail, which is 70 mm to the straight line AB in the wheel rim segment. The rolling circle consists of all the contact points around the wheel, the diameter of which is defined as the wheel diameter. Point E is the measured point of the 1D-LDT, which is in the straight line DF. The distance *d_EC_* between the measured point and the contact point is known by the layout of the three 1D-LDTs, the typical value of which is 47 mm. It should be noted that *D_m_* in Equation (6) is not the wheel diameter, but the diameter of the measured circle. To measure the wheel diameter, the distance *h_EC_* between the measured point and contact point should be determined. Thus, the SLVT is used to measure the wheel tread profile, the measurement principle of which is described in the reference [17].

The two-dimensional coordinates of the wheel tread profile can be obtained by the SLVT. After that, the straight line AB can be obtained by fitting; then, the coordinate of contact point C is determined by the distance to the line AB. As the distance *d_EC_* is known, the coordinate of the measured point can be accurately determined. The difference between the y-coordinates of the two points is *h_EC_*, and the wheel diameter *D* can be obtained by

(7) $$D=D_{m}{+2h}_{EC}.$$

## 3. Measurement Error Analysis

From Equations (6) and (7), the diameter can be calculated simply by measuring the special distances of *l*_1_, *l*_2_, *h*, and *h_EC_*. *h_EC_* is measured by the SLVT, and the measurement error can be less than 0.1 mm [18], which results in an error for measuring the diameter of less than 0.2 mm. There are four other primary factors that can influence the diameter measurement accuracy: The S-shaped motion of the wheel, the measurement error of 1D-LDT, the positioning error of the wheel, and the rail deformation caused by the wheel-rail interaction force. The measurement errors caused by the S-shaped motion of the wheel and the rail deformation are systematic errors for which it is possible to compensate. The measurement errors caused by the measurement error of 1D-LDT and the positioning error of the wheel are random errors, for which it is not possible to compensate. In the following section, these factors are analyzed and simulated in theory, where *L* is set to 480 mm, *h* is set to 80 mm, *α* is set to 60°, and *l* is set to 285 mm.

### 3.1. Measurement Error of the Transducer

**Figure 6:**
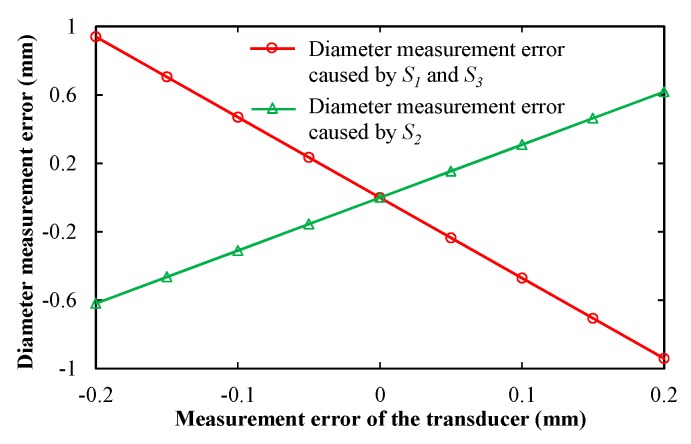
Diameter measurement error caused by the measurement error of the transducer.

Figure 6 shows the theoretical stimulation results of the diameter measurement error caused by the measurement errors of *S*_1_ and *S*_3_, as well as *S*_2_. In Figure 6, the measurement error of the transducer is set between −0.2 and 0.2 mm at intervals of 0.05 mm. In fact, the measurement error of the transducer can be less than 0.05 mm [19]; thus, the diameter measurement error is less than 0.24 mm caused by the measurement errors of *S*_1_ and *S*_3_. For *S*_2_, it is less than 0.15 mm.

### 3.2. Positioning Error of the Wheel

**Figure 7:**
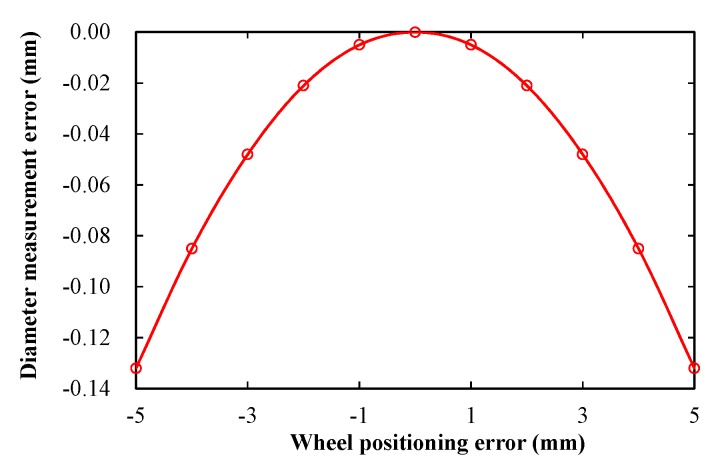
Diameter measurement error caused by the positioning error of the wheel.

Figure 7 shows the theoretical stimulation result of the diameter measurement error produced by the wheel positioning error, where the positioning error is zero when S_2_ is below the lowest point of the wheel. According to Equation (6), if the positioning error is within 5.0 mm, the diameter measuring error is less than 0.13 mm. Actually, it is easy for the wheel positioning error being controlled to be less than 1 mm based on the sum of the outputs of *S*_1_ and *S*_3_; thus, the diameter measurement error caused by the wheel positioning error is negligible. 

### 3.3. S-Shaped Motion of the Wheel

**Figure 8:**
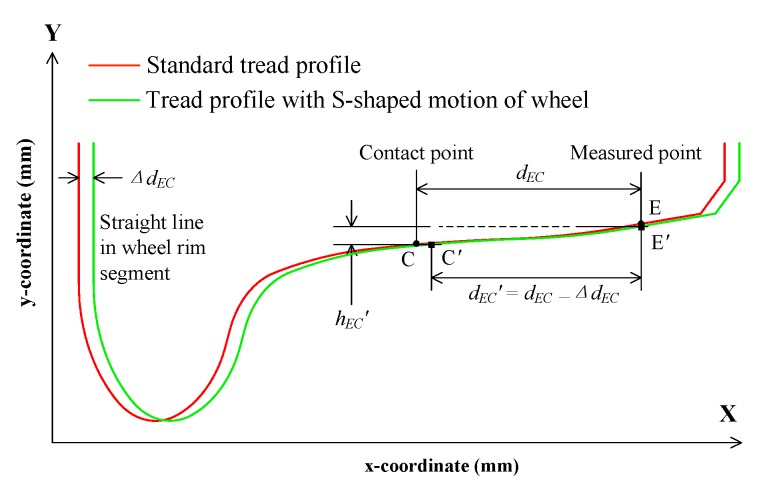
Tread profile with the S-shaped motion of wheel.

Figure 8 shows the tread profile with the S-shaped motion of the wheel, wherein the standard tread profile is obtained by measuring the standard wheel using the SLVT. Owing to the S-shaped motion of wheel, the contact point C has changed to C′, and the measured point E has changed to E′ in the Y-axis direction. In Figure 4, the S-shaped motion displacement Δ*d_EC_* can be measured by the difference between the two straight lines in the wheel rim segment. Thus, the corrected distance *d_EC_′* between the contact point and the measurement point is expressed by

(8) $$d_{\mathrm{EC}}{}^{\prime }=d_{\mathrm{EC}}-\Delta d_{\mathrm{EC}}.$$

Here, *d_EC_′* is used to search the measured point in the tread profile with the S-shaped motion of the wheel, and the corrected distance *h_EC_′* can be determined. In this way, the influence of the S-shaped motion of the wheel on the diameter measurement can be eliminated in theory.

### 3.4. Rail Deformation

**Figure 9:**
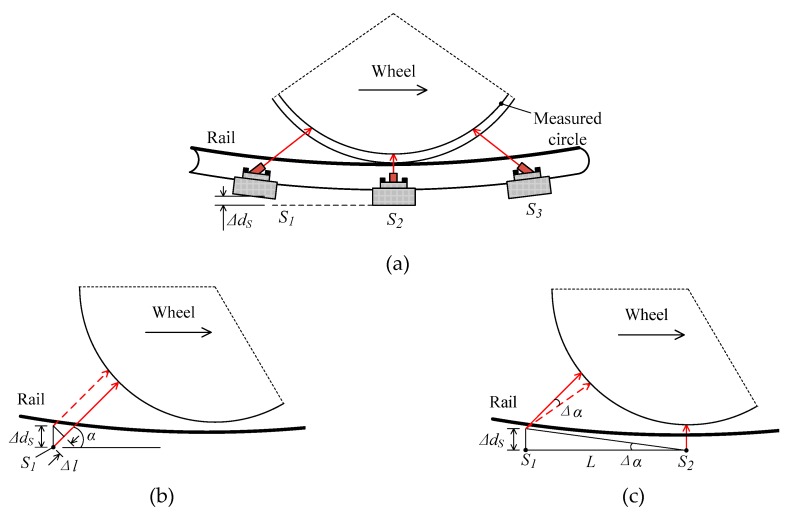
Influence of rail deformation: (**a**) Schematic diagram of rail deformation; (**b**) change in the measured distance of the sensor; (**c**) change in the direction of the laser beam.

Rail deformation caused by the wheel-rail interaction force is inevitable. As shown in Figure 9a, there is a distance Δ*d_S_* in the vertical direction of *S*_1_ to *S*_2_ and *S*_3_ to *S*_2_ because of rail deformation when the train passes thorough the transducers; thus, the measured distance error Δ*l* of the transducer and the direction error Δ*α* of the laser beam are produced. From Figure 9b, Δ*l* can be expressed by

(9) $$\Delta l=\Delta d_{S}\sin\alpha .$$

From Figure 9c, Δ*α* is obtained by

(10) $$\Delta \alpha =\arctan\left(\frac{\Delta d_{S}}{L}\right).$$

To analyze the effect of rail deformation on diameter measurement, Δ*d_S_* is a key parameter and should be determined. A vehicle-track, rigid-flexible coupling model was established using the combination of multi-body dynamics and finite element methods, and rail deformation was analyzed at low and high train speeds. 

**Figure 10:**
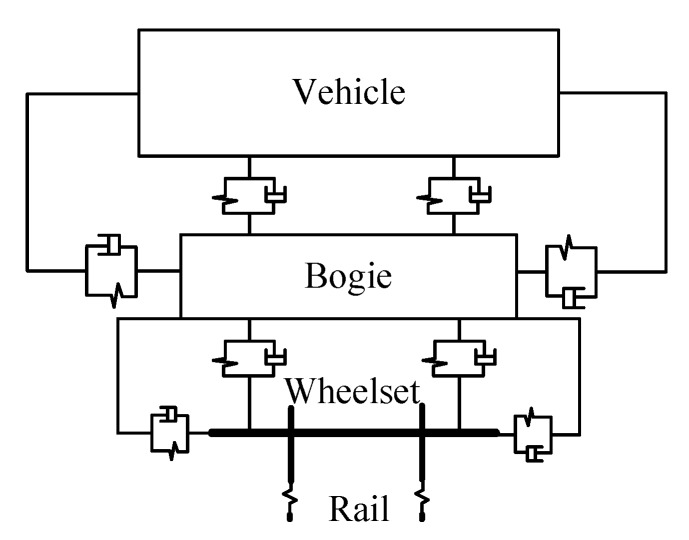
Dynamic model of the vehicle system.

Figure 10 shows a dyna-mic model of the vehicle system, wherein the vehicle and the track constitute the vehicle-track coupling dynamic system. The vehicle model is a multi-body system composed of one body, two frames, four wheels, and the primary and secondary suspension systems. The finite element analysis software Abaqus CAE [20], which is developed by Dassault Systems and it is a complete solution for finite element modeling, visualization, and process automation, was used to produce the flexible rail at first, and the elastic part was then generated using the software Simpack [21], which is a general multi-body simulation software enabling analysts and engineers to simulate the non-linear motion of any mechanical or mechatronic system. The values of the parameters used in the simulation are listed in Table 1. Using a combination of the structural parameters and suspension parameters of the vehicle system, the vehicle-track rigid-flexible coupling model was established.

**Figure 11:**
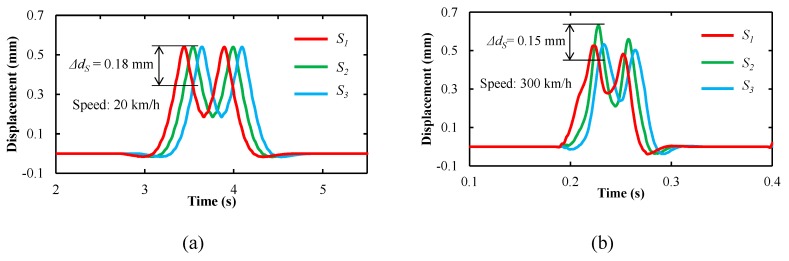
Simulation results: displacements of the transducers at the speeds of (**a**) 20 km/h and (**b**) 300 km/h.

Displacement-time curves of *S*_1_, *S*_2_, and *S*_3_ at the different speeds of the train using the vehicle-track rigid-flexible coupling model are shown in Figure 11. The displacement-time curve of the transducer can represent rail deformation, as it is fixed on the rail. From Figure 11a,b, rail deformation was different at low and high speeds. At a low speed of 20 km/h, the distance Δ*d_S_* was 0.18 mm. At a high speed to 300 km/h, it was 0.15 mm. From Equation (9), the measured distance errors Δ*l* were 0.16 and 0.14 mm at speeds of 20 and 300 km/h, respectively. Thus, the diameter measurement errors are ±0.75 and ±0.66 mm according to Figure 6.

**Figure 12:**
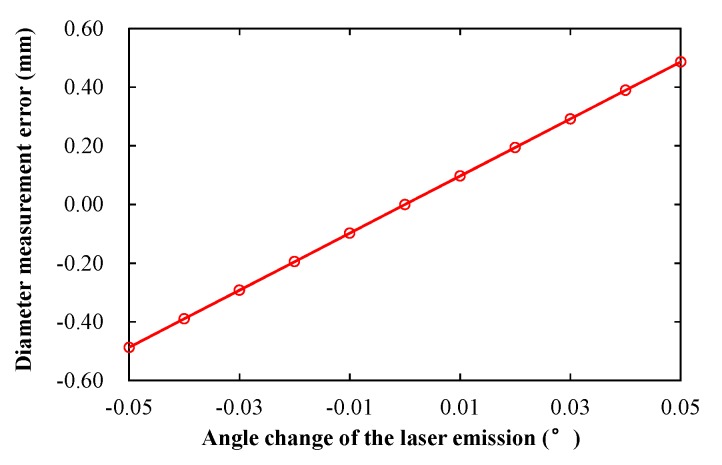
Diameter measurement error caused by the angle change of the laser emission.

Figure 12 shows the diameter measurement error caused by the angle change of the laser emission. According to Equation (10), Δ*α* were 0.019° and 0.017° at low and high speeds of the train, respectively. Thus, the diameter measurement errors are ±0.19 and ±0.17 mm.

**Figure 13:**
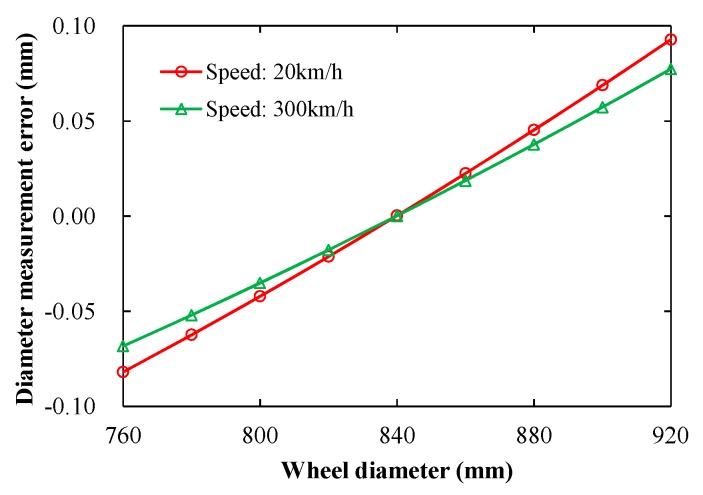
Diameter measurement error caused by an angle change of the laser emission.

The diameter measurement errors caused by the rail deformation are systematic and can be compensated by the comparative measurement with a standard wheel whose diameter is known. Thus, Equation (6) should be expressed as

(11) $$\begin{array}{l} D_{m}=D_{m}{}^{\prime }+\frac{\left(l_{1}{}^{\prime }-l_{1}\right)\left(L^{2}\sin\alpha -h^{2}\sin\alpha -2Lh\cos\alpha +hl_{1}{}^{\prime }+hl_{1}-l_{1}{}^{\prime }l_{1}\sin\alpha \right)}{2\left(l_{1}{}^{\prime }\sin\alpha -h\right)\left(l_{1}\sin\alpha -h\right)}+\\ \;\frac{\left(l_{2}{}^{\prime }-l_{2}\right)\left(L^{2}\sin\alpha -h^{2}\sin\alpha -2Lh\cos\alpha +hl_{2}{}^{\prime }+hl_{2}-l_{2}{}^{\prime }l_{2}\sin\alpha \right)}{2\left(l_{2}{}^{\prime }\sin\alpha -h\right)\left(l_{2}\sin\alpha -h\right)} \end{array}$$

where *D_m_*′ is the standard diameter of the measured circle in the standard wheel, *l*_1_′ and *l*_2_′ are the output distances of *S*_1_ and *S*_3_ to measure the standard wheel, respectively. Figure 13 shows the simulation results based on the comparative measurement, where the diameter of the standard wheel is 840 mm. For both low and high speeds, the diameter measurement error is less than 0.1 mm, with the wheel diameter being in a range of 760–920 mm. Thus, the influence of rail deformation on diameter measurement can be greatly reduced.

## 4. Field Experiments 

**Figure 14:**
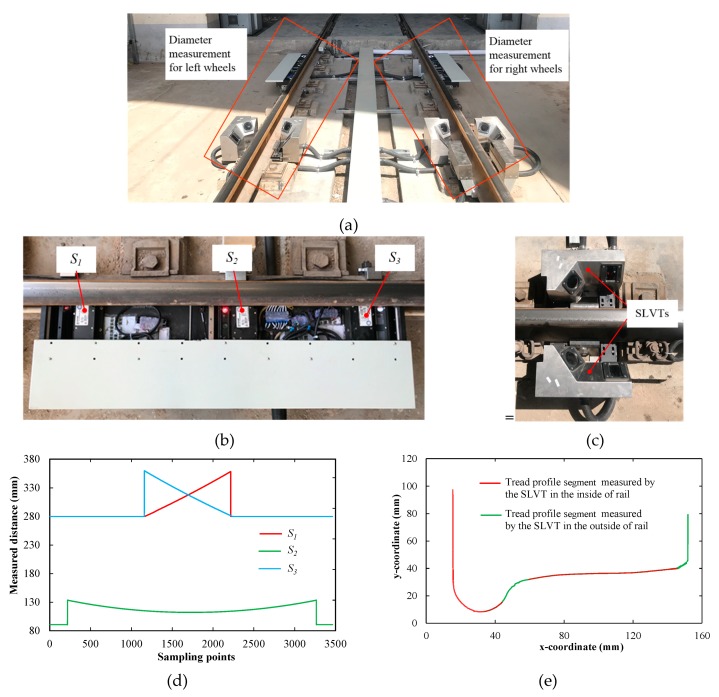
Images of the field experiment and real outputs of the transducers: images of (**a**) developed measurement system, (**b**) one-dimensional laser displacement transducers (1D-LDTs), and (**c**) structural light vision transducers (SLVTs); outputs of (**d**) three 1D-LDTs and (**e**) two SLVTs.

The corresponding system for dynamically measuring the train wheel diameter based on the aforementioned method was developed, and was installed in the storage line of Yinchuan railway station vehicle depot, China, as shown in Figure 14a. Figure 14b,c show images of the 1D-LDTs and SLVTs, respectively. Here, three developed 1D-LDTs and two developed SLVTs were used. In Figure 14b, the three 1D-LDTs were installed in a box, which was mounted on the standard rail. In the box, *S*_1_ and *S*_3_ were mounted symmetrically, 480 mm away from *S*_2_. The lasers of *S*_1_ and *S*_3_ were at an angle of 60°. In addition, *S*_2_ had a measurement range of 40 mm, and the start measurement distance was 90 mm. For *S*_1_ and *S*_3_, the measurement range was 80 mm, the start measurement distance was 280 mm. A grating ruler (LG-50, accuracy: 0.1 μm, resolution: 50 nm) was used to calibrate the 1D-LDTs. The results showed that the measurement accuracy was 20 μm for *S*_2_, and that was 15 μm for *S*_1_ and *S*_3_. In Figure 14c, the SLVT was mainly composited by a camera (acA1600-20gm, Basler, pixel: 1626 × 1236), a lens (M1620-MPW2, Computer, focal length: 16 mm), and a line laser (EL65L200IG9, Elite, power: 200 mW), which was installed in an individual box. Two SLVTs, mounted on both sides of the standard rail, were used to produce a complete tread profile. When the train wheel passed through the measurement system, the three 1D-LDTs sampled synchronously with a frequency of 5 kHz, and the two SLVTs were triggered by a photoelectric switch. The real outputs of the three 1D-LDTs and two SLVTs are shown in Figure 14d,e respectively.

To verify the performance of the developed measurement system, the diameters of four wheels with a small roundness error in a train car (ID: 350122), which passed through the measuring system three times in a month, were dynamically measured. The experimental information and results are shown in Table 2.

Table 2 shows that our system has a good performance in repeatability error. The repeatability error of wheels 2 and 4 is 0.09 mm, and the maximum repeatability error is 0.27 mm. In addition, the diameter of wheel 4 was measured by an underfloor wheelset lathe (U2000-150, hegenscheidt, diameter measurement accuracy: 0.1 mm); the result was 889.38 mm. Thus, the deviation between our system and the lathe is 0.22 mm. Consequently, the repeatability and measurement errors of the proposed system are less than 0.3 mm, which meets the requirement of Chinese high-speed railway operations.

## 5. Conclusions

In this paper, a high-precision method using three 1D-LDTs for dynamically measuring wheel diameters was first proposed. The factors that influenced measurement accuracy were analyzed. As a main factor, the rail deformation caused by the wheel-rail interaction force at low (20 km/h) and high (300 km/h) speeds was determined based on the combination of the multi-body dynamics and finite element methods, and the effect of rail deformation on measurement accuracy was greatly reduced by a comparative measurement. A corresponding measurement system was developed. The performance of the proposed measurement system was verified by field experiments. The experimental results demonstrate that the repeatability and measurement errors of the system are less than 0.3 mm. Future work will focus on high-speed dynamic diameter measurements for high-speed trains.

## Figures and Tables

**Table 1:** Parameters used in simulation.

Parameters	Values	Parameters	Values
Mass of car body	3.43 × 10^4^ kg	Pitch moment of inertia of bogie	1205 kg∙m^2^
Mass of bogie	2235 kg	Yaw moment of inertia of bogie	2792 kg∙m^2^
Mass of wheel	1200 kg	Stiffness of primary suspension system	9.198 × 10^5^ N/m
Roll moment of inertia of car body	9.25 × 10^4^ kg∙m^2^	Stiffness of secondary suspension system	1.33 × 10^5^ N/m
Pitch moment of inertia of car body	1.756 × 10^5^ kg∙m^2^	Damping of primary suspension system	1 × 10^4^ N∙s/m
Yaw moment of inertia of car body	1.728 × 10^5^ kg∙m^2^	Damping of secondary suspension system	8400 N∙s/m
Roll moment of inertia of bogie	1846 kg∙m^2^		

**Table 2:** Experimental information and results.

Number	Data and Time	Train Speed (km/h)	Temperature (°)	Wheel No.			
				1 (mm)	2 (mm)	3 (mm)	4 (mm)
1	18:21, 4 June, 2019	3.1	26.6	891.10	881.64	887.67	889.12
2	18:22, 5 June, 2019	3.5	28.4	890.68	881.45	887.59	889.09
3	23:08, 22 June, 2019	3.9	20.3	891.22	881.45	887.32	889.27
Average value (mm)				891.00	881.51	887.53	889.16
Repeatability error (mm)				0.27	0.09	0.17	0.09
Result of the lathe (mm)							889.38
Deviation (mm)							0.22
